# Barriers and Facilitators for Implementing Digital Interventions for Anxiety and Depression in Latin America: A Scoping Review

**DOI:** 10.3390/ijerph22040628

**Published:** 2025-04-17

**Authors:** Bárbara Battistotti Vieira, Léa Savoy, Kathya Acuña Luna, Antoine Flahault, Jennifer Hasselgard-Rowe

**Affiliations:** 1 Global Studies Institute, University of Geneva, 1205 Geneva, Switzerland; lea.savoy@etu.unige.ch (L.S.); kathya.acuna@etu.unige.ch (K.A.L.); 2 Faculty of Medicine, Institute of Global Health, University of Geneva, 1211 Geneva, Switzerland; antoine.flahault@unige.ch (A.F.); jennifer.hasselgard-rowe@unige.ch (J.H.-R.)

**Keywords:** digital health interventions, Latin America, mental health

## Abstract

Mental health disorders have a high prevalence in Latin America (LATAM), with an estimated 6.7% of the population suffering from anxiety and 4% from depression. Digital mental health interventions (DMHIs) have been implemented to address these issues. However, there has yet to be a clear picture of these interventions in the region. This scoping review aims to analyze DMHIs’ characteristics and the barriers and facilitators for their implementation in five LATAM countries (Brazil, Chile, Colombia, Mexico, and Peru). To achieve this goal, four databases (PubMed, APA PsycNet, Scielo, and LILACS) were searched using relevant search terms in English, Spanish, and Portuguese. A total of 484 references were identified and narrowed down to 15 articles included in the final analysis. The studies mostly consisted of RCTs and mixed-methods studies. Most of the DMHIs were designed for individuals, with a focus on targeted communication and personal health tracking. Interventions targeting healthcare professionals were less common, primarily focusing on decisional support. The most prominent barriers to the successful implementation of DMHIs included insufficient healthcare professional training (40%) and resistance from clinicians and organizational culture (40%), while common facilitators included easy access to the interventions (46.7%) and increased training and support for clinical teams (46.7%). Positive outcomes were reported in terms of both effectiveness (33.3%), with many interventions showing results equal to or better than traditional methods (33.3%), and patient satisfaction (33.3%).

## 1. Introduction

Digital mental health interventions (DMHIs) are increasingly recognized as a valuable approach for addressing barriers to in-person mental health (MH) care, such as stigma, accessibility, cost, and geographic limitations [1]. However, there is a paucity of research on the use of DMHIs for prevention and treatment in Latin America (LATAM), despite the widespread use of mobile technology in the region [2]. MH disorders, including depression and anxiety, are highly prevalent in LATAM [3]. Despite the significant burden of MH issues, the region faces a severe shortage of MH professionals, with only 8.2 MH workers per 100,000 people in Central America, Mexico, and the Latin Caribbean and 23.9 per 100,000 in South America—both figures are starkly lower than the 283.1 MH workers per 100,000 people in North America. MH funding is equally limited, with only 3% of total government health expenditure allocated to MH across the Americas. This is even lower in Latin America, where it is just 1.8% [4]. It is estimated that 74% of individuals do not receive the care they need in LATAM [5].

Several factors contribute to poor MH outcomes in the region, including structural challenges like limited investment in MH care and insufficient political will for reform [5]. On an individual level, stigma, low MH literacy, and financial constraints also impede access to care [6]. Social inequalities, such as unemployment, poor working conditions, and inadequate housing, were further exacerbated during the COVID-19 pandemic, intensifying MH issues in the region [7,8].

Acknowledging LATAM’s cultural and geographical diversity, this review focuses on five countries in the region: Mexico, Colombia, Brazil, Peru, and Chile. They were selected since, combined, they host almost three-fourths of LATAM’s population [9]. These countries also have national digital health strategies and open data policies [10]. Additionally, depression and anxiety remain highly prevalent in the selected countries. In 2021, the prevalence of anxiety was 9% in Brazil, 5.6% in Colombia, 4.5% in Mexico, 7.2% in Peru, and 6.6% in Chile. The prevalence of depression was 4.4% in Brazil, 2.3% in Colombia, 4.4% in Mexico, 2.7% in Peru, and 4.6% in Chile during the same year [11]. The prevalence of anxiety and depression in other LATAM countries followed the same trajectory. In Argentina, anxiety reached 6.3% and depression reached 3.2%, and in Panama, they reached 4.7% and 3.5%, respectively.

The MH policy landscape in LATAM significantly influences the availability and relevance of DMHIs. Common trends include a focus on community-based care, deinstitutionalization, reduced reliance on psychiatric hospitals, prioritization of primary healthcare, and training general practitioners to address MH issues. Mexico reformed its Health Law in 2022 and adopted the WHO’s MH Gap Action Programme (mhGAP) to train primary care providers [12]. Similarly, Peru implemented the WHO’s mhGAP and enacted a National MH Law, establishing over 100 community MH centers [13]. Chile adopted a National MH Plan (2017–2025) [14], while Colombia’s 2018 National MH Policy emphasizes prevention and community support alongside mhGAP adoption [15]. Brazil established its Psychosocial Care Network (RAPS) in 2011, integrating MH services with primary care [16]. These policies reflect a growing recognition of MH as a public health priority, with tailored approaches addressing local needs in each country.

Many low- to middle-income countries require institutional support for developing and implementing DMHIs, as these efforts demand significant resources and expertise [17]. In the wake of the COVID-19 pandemic, digital health has become even more crucial for expanding healthcare access, particularly in integrating MH services into digital platforms [18]. Despite this growing importance, there is currently no post-COVID-19 pandemic research specifically examining DMHI implementation in LATAM, particularly regarding barriers and facilitators. Addressing this gap is essential for improving service provision and guiding future interventions. Given the high prevalence of MH disorders and the shortage of services in LATAM, this scoping review aims to characterize existing DMHIs in the region, with a focus on depression and anxiety. Specifically, this study seeks to answer the following research question: What is the current landscape of DMHIs in LATAM—including their types, uses, and target population—and what barriers and facilitators influence their implementation? To address this, we examine DMHIs using the WHO’s Classification of Digital Interventions, Services, and Applications in Health (CDISAH), further detailed in the methodology section [19]. This study contributes to existing literature by providing a comprehensive analysis of DMHIs in LATAM.

## 2. Materials and Methods

### 2.1. Information Sources and Search

**Figure 1:**
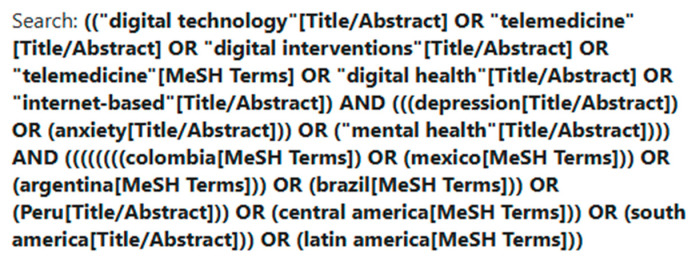
Screenshot of the English search equation in PubMed.

As part of this scoping review, a search was performed to find potentially relevant articles using the following databases: PubMed, APA PsycNet, SCIELO, and LILACS. Searches were conducted in English, Spanish, and Portuguese to ensure that we were obtaining articles with authorship from the global north and global south. The search strategy included various synonyms for digital technology, MH (and specifically depression and anxiety), and the list of countries we wanted to focus on in LATAM. Argentina was originally chosen for this study, but no articles from the country were found in the database search. Chile was later included in the analysis instead. All search results were then exported to a joint Microsoft Excel file, and duplicates were removed automatically. Additionally, a manual duplicate exercise was conducted to detect duplicates that included the same article translated into other languages and any other duplicates that had not been identified. Relevant studies included intervention practices using digital technology for MH within at least one LATAM country. The search equations used in PubMed (see Figure 1), APA PsycNet, Scielo, and LILACS in all three languages are included in Appendix A (Table A1).The following table (Table 1) displays the search terms used in our equations.

### 2.2. Eligibility Criteria

Only peer-reviewed studies applied in real-world settings in LATAM and intervention studies were included. Discussion pieces, reviews, design proposals, or studies assessing impact factors related to but not directly involving an intervention were excluded. For instance, we excluded discussions related to COVID-19’s impact as a driving force on DMHIs without describing an intervention implemented during the pandemic, which was a significant proportion of our preliminary results, as our analysis required a focus on studies that examined an actual DMHI implementation. Articles discussing MH disorders that did not include or did not differentiate between anxiety and depression were excluded. Articles where neither the abstract nor keywords in the abstract were available were automatically excluded. Articles where the full paper was not accessible to any of the researchers were not considered. Finally, only articles published between 2014 and 2024 were included.

### 2.3. Data Collection and Analysis

**Figure 2:**
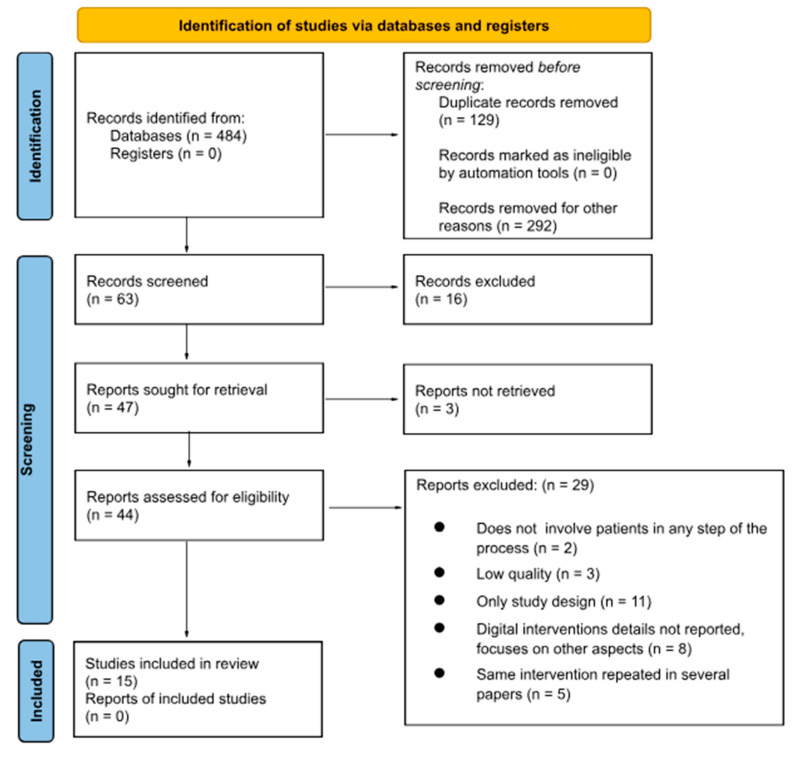
PRISMA 2020 flow diagram.

The study selection process followed the PRISMA-ScR guidelines and is illustrated in the PRISMA flow diagram (see Figure 2). This scoping review was not registered. Each research member evaluated the article, abstract, and then full text (if relevant) of the articles in the database search by codifying each article as low priority, medium priority, or high priority. Low-priority articles labeled by the research team were removed based solely on the title as they demonstrated a lack of relevance to the researched topic. Abstracts of medium-priority articles were read and removed if the study did not have data (i.e., discussed study protocol or ongoing data collection) or if depression or anxiety were not included as observed disorders. High-priority articles were read, and data were extracted to synthesize sample description, study design, type of intervention used, intervention description, and primary outcome. From this, high-priority articles were discussed amongst research members and evaluated if they fit the selection criteria article for this scoping review. After the final article selection and based on the defined research question, repeating themes and categories were codified for each article. The discrete label categories were the following, with multiple labels possible for each category: country of origin, MH disorder (i.e., depression and/or anxiety), tech type (mobile apps or websites), barriers, facilitators, evaluation metrics (method used by each intervention to report their results or impact), and target population.

To analyze the DMHIs being implemented in LATAM, this review used the WHO’s CDISAH (2023) as a framework. This classification provides a systematic approach to better understand the connection between healthcare challenges and DHIs. It establishes a foundational framework for describing DHIs to improve research, coordination of various key stakeholders, and policy-making. DHIs are categorized and organized into communication platforms, data services and applications, platforms for learning, systems of transaction, and utility services as a way to support global or national healthcare initiatives. This classification of “types” enables key stakeholders to clearly understand the connection between health technologies and reducing challenges in healthcare.

## 3. Results

### 3.1. Descriptive Analysis

A total of 15 articles were included in the final analysis: nine from PubMed, five from LILACS, and one from PsycNet, while no results were found in the Scielo database. Geographically, these publications originated from five countries: four from Peru, three from Brazil, three from Colombia, one from Mexico, and one from Chile. Three DMHIs were implemented across two countries simultaneously (Brazil and Peru; Colombia and Chile; Colombia and Mexico). Regarding the target populations, 60% of the articles focused on the general adult population.

Additionally, two articles targeted adults, children, and teenagers, while another two focused exclusively on children and teenagers. One study centered on university students, and another targeted newly graduated physicians working in rural areas. Analyzing the publication trends based on the periods before and after the onset of the COVID-19 pandemic reveals distinct shifts in activity. The articles can be divided into two groups: pre-pandemic and post-pandemic (2020 onwards). There were relatively fewer publications during the pre-pandemic period, with only two articles published in 2018. There is a noticeable increase in publication activity following the pandemic’s onset. Starting in 2020, the number of articles published each year rose significantly, with a peak in 2021, which saw four publications. The trend continued with consistent outputs in 2022 and 2023, and a notable surge in 2024, with the highest count of three articles in that single year alone.

### 3.2. WHO’s CDISAH

Varied use of technology types was identified (see Table 2). The most frequently utilized technology was websites, which accounted for 26.7% (4 out of 15) of the interventions. This was followed by telehealth (online communication) and apps, each used in 20% (3 out of 15) of the cases. Mixed online media was applied in 13.3% (2 out of 15) of the interventions, matching the usage rate of chatbots, also at 13.3%, tools that are usually powered by artificial intelligence (AI). Finally, electronic databases were the least used, featuring in just 6.7% (1 out of 15) of the DHIs. These percentages highlight a preference for websites and telehealth methods over other DHI modalities.

The vast majority of the DMHIs (80%) were classified as interventions for persons (12 out of 15), while only three were interventions for healthcare professionals. Among interventions aimed at persons, the most common strategy was targeted communication, used in five cases, which accounts for 33.3% of the interventions. This was followed by personal health tracking, featured in three interventions, representing 20%. Telemedicine was employed in two cases, or 13.3%, with an additional intervention combining both targeted communication and telemedicine. Another intervention integrated telemedicine with data management, which represents 6.7%. Additionally, there was one instance of on-demand communication with persons, accounting for 6.7%. For healthcare professionals, the interventions focused more on decision support and data management. Specifically, there was one intervention dedicated to healthcare provider decision support (6.7%) and another that combined data management with decision support (6.7%).

### 3.3. Barriers and Facilitators

The literature revealed that many barriers and characteristics are common throughout the selected countries (see Table A2). Notably, with only one exception (article 7) [26], all interventions were focused primarily on treatment over prevention and were developed predominantly in the countries of implementation by local researchers. However, depression treatment can be considered a preventive measure for other diseases, such as dementia [35].

The most commonly mentioned barriers were “More training needed” (40%) (article 3, 7, 10, 12, 13, 14) and “Lack of clinicians involvement or management and culture organization resistance” (40%) (article 1, 8, 12, 13, 14, 15). These were followed by “High dropout rate” (33.3%) (article 4, 5, 6, 7, 11) and “No time-availability” (26.7%) (article 8, 15). Santa-Cruz et al.’s intervention in Peru elaborates on their issues regarding a high dropout rate, mentioning the possibility that the method of re-assessment using phone calls vs. the original screening chatbot used in the study may have influenced the high percentage of participants which could not be contacted for follow-up [29]. Some of the reasons considered include “theft or loss of their phones, cancellation of phone services, or disinterest in picking the call for re-assessment”. Illustrating cultural organization resistance to the intervention tools, Martínez et al.’s study explains, “The use of the SEHR … was not integrated into the daily routine of the participating physicians, and a limited time scheduled for this activity had to be negotiated with the local authorities. Moreover, the primary care teams were working in a context of high patient demand and regular staff turnover. Thus, registering patient information in the SEHR may have been experienced as burdensome by some of the physicians, partly explaining the low use of this system” [26].

Many studies emphasized resistance to integrating digital tools into their routine workflows, often attributed to limited proficiency with the tools and the perception of DHIs as an additional workload. This can be exacerbated by the development of these tools without adequate consultation or involvement of healthcare workers during the design phase. On a smaller scale, technological barriers, like the need to ensure data storage safety, data privacy, and anonymity for participants (article 5, 6, 13); the lack of internet access of some of the target populations (article 10, 12); and the technology being too complicated (article 3, 5) or not culturally appropriate for the patient users (article 2, 7, 8), persist. As an example of an intervention not being culturally appropriate, participants’ feedback from the study conducted by Martinez et al. indicated the need to improve the intervention by increasing its levels of customization, specifically citing that participants wished it included more interaction with team members through activities, and diversifying the questions, focusing more on outside-of-school activities [26]. Furthermore, this intervention had very different success outcomes in Colombia compared to Chile, suggesting that what might be appropriate for one population will need to be readapted depending on the setting and context. Santa-Cruz et al.’s study comments on the lack of internet access as follows: “...although internet users have increased in Peru during the pandemic, almost half of the population accessing ChatBot-Juntos initially found out about the tool in the community and not through social media platforms, suggesting that social media does not reach the entire population and access to technology may still be limited” [29].

Regarding the facilitators, 46.7% (article 2, 3, 4, 5, 9, 10, 11) mentioned the DHI was easy to access, which increased the availability of MH services for patients. As an example, Benjet et al.’s study cites, “College students are in some ways an ideal population for i-interventions, given that they are required to have some degree of internet access and literacy to complete their coursework… virtually all students used the internet, with 90% accessing it in their homes and the rest either at the university, in public spaces, or in the homes of others” [28]. Additionally, during the interventions, clinical teams received increased training and support (46.7%) (article 6, 7, 8, 12, 13, 14, 15), even though this increased training was still perceived as insufficient to cover the learning curve of technologies, as discussed earlier. Gómez-Restrepo exemplifies it best, mentioning they “addressed some of the identified barriers for detection of mental health conditions, such as low awareness about these conditions among GPs and patients and lack of training among GPs about how to address these conditions… We repeatedly explained and underscored the importance of embracing mental health as part of healthcare via constant feedback, retraining and consultation, not only for the GPs but also for the institutions, to align the multiple actors with the objectives of the model. Through regular meetings in which we offered feedback and engaged in case-based discussion, as well as site-specific reports of results, we observed an improvement in the confirmation rates of diagnoses” [34].

Furthermore, most articles concluded that DHIs had positive outcomes, reporting equal or better initial results than traditional methods (33.3%) (article 1, 3, 4, 5, 7). For example, Araya and colleagues (article 3) describe that the proportion of Brazilian participants with a reduction in their PHQ-9 (Patient Health Questionnaire-9 is used for screening, diagnosing, monitoring, and measuring the severity of depression) score of at least 50% at a 3-month follow-up assessment was 40.7% in the digital intervention group vs. 28.6% in the enhanced usual care group (difference, 12.1 percentage points (95% CI, 5.5 to 18.7); adjusted odds ratio (OR), 1.6 (95% CI, 1.2 to 2.2); *p* = 0.001) [22]. Additionally, the proportion of Peruvian participants with a reduction in their PHQ-9 score of at least 50% at a 3-month follow-up visit was 52.7% in the digital intervention group vs. 34.1% in the enhanced usual care group (difference, 18.6 percentage points (95% CI, 9.1 to 28.0); adjusted OR, 2.1 (95% CI, 1.4 to 3.2); *p* < 0.001) [22]. Additionally, most articles reported high or increased patient satisfaction (33.3%) (article 1, 6, 7, 9, 12). Moretti and colleagues (article 6) provided participants with an online satisfaction survey [25]. The survey indicated that overall satisfaction in samples 1–3 was high, with the mean across all questions being 4.5 (SD 0.76) on a 5-point Likert scale (1 = strongly disagree through 5 = strongly agree); specifically, 75% of respondents marked a 5 for the platform’s useful information on personal growth and 70% of respondents marked a 5 for the platform’s relevance of materials, visual graphics, and usefulness for feeling better [25]. Martínez, Rojas, and others (article 12) also reported satisfaction on a 7-point Likert scale, with satisfaction with psychological care displaying a significant difference between the groups (Wilcoxon rank-sum test *p* value = 0.04), with the RCDC (Remote Collaborative Depression Care Program) intervention scoring higher (compared to the control group who received enhanced usual care) [31].

On the logistics side, a couple of interventions proved to be less costly than traditional methods (26.7%) (article 5, 9, 10, 11). Santa-Cruz and colleagues (article 10) incorporated a “chatbot” feature in their DMHI; these digital conversational agents offer an intelligent and automated system for detecting and responding to immediate mental health needs [29]. For Santa-Cruz and colleagues, the chatbot improves productivity, uses resources efficiently, and increases access, thus reducing cost [29].

## 4. Discussion

“Lack of clinician involvement and cultural organization resistance” emphasizes engaging healthcare providers and administrators early in the design and implementation process. When clinicians feel excluded from the development of DHIs, they are less likely to view these tools as relevant to their work and contribute to organizational resistance, where DHIs are seen as an additional burden rather than a supportive resource. Cultural factors within organizations, such as hierarchical decision-making and resistance to change, without strong leadership advocating for digital innovation, further exacerbate this issue. While these findings align with those of a previous MH internet-based prevention review, which highlighted challenges in engaging participants, retaining them for sufficient durations, and ensuring adherence to protocols [36], they are not the main identified barriers. Other identified barriers that are echoed in another systematic review are infrastructure, lack of equipment, and technology gaps [2].

By reducing geographic and logistical barriers, DHIs expand access to MH services. This is a critical enabler of success, especially for underserved populations in LATAM. These tools can offer flexibility, enabling patients to engage at their convenience. Furthermore, many DHIs are designed to be user-friendly and accessible across devices, further enhancing their reach and usability. Although one of the most frequent barriers was “more training needed”, increased training and support provided to clinical teams during interventions still helped. These efforts familiarize healthcare workers with the technology, improving their confidence and competence. However, there is still a significant gap in digital proficiency as the training was either insufficient or technical. This reflects the duality of these DHIs: though training is provided, it is still not enough to implement these interventions adequately.

In the short term, expanding clinician training programs is a highly actionable step, as it directly improves digital proficiency and enhances the successful adoption of DHIs. Training should go beyond technical skills and include practical applications tailored to real-world clinical settings, as seen in Brazil’s Telemedicine University Network (RUTE) [37], which has successfully integrated digital health training into medical education. Additionally, providing financial incentives or protected time for clinicians to engage in DHI training can facilitate adoption. Mid-term solutions should focus on strengthening institutional infrastructure, such as ensuring healthcare facilities have adequate digital tools and internet connectivity, similar to initiatives in India’s National Telemedicine Service (eSanjeevani) [38], which improved rural healthcare access. Long-term strategies will require significant government investment to ensure equitable access. Rwanda’s national broadband initiative [39] demonstrates how sustained policy commitment can significantly enhance digital healthcare reach. By aligning these different strategies, policy-makers can create a sustainable framework for DHI implementation, maximizing impact while addressing the key barriers identified by this review.

This scoping review concentrated on publications addressing anxiety and depression due to their comorbidity, as they use similar treatment methods and interventions [40]. In contrast, other mental health disorders involve various approaches to diagnosis, severity, and treatment [41]; this is different from similar scoping reviews in the field [36]. This focused approach allows for a more effective framework that highlights the potential for developing DHIs for comorbid mental health disorders.

### Limitations of the Study

This study faced constraints related to the search process, including potential language biases, restricted access to certain databases, and the availability of relevant studies. The decision to focus narrowly on depression and anxiety may have resulted in the exclusion of other important MH disorders that could have provided a more comprehensive understanding of the topic. The review specifically concentrated on interventions directly involving patients, thereby excluding potentially relevant initiatives directed toward other key stakeholders, such as healthcare workers, improvements in healthcare record storage systems, or interventions targeting organizational and systemic actors. Additionally, although many articles describe the same barriers and facilitators, many of these points (i.e., patient satisfaction or reduction in symptoms) are measured differently across the selected studies. These exclusions may have limited the breadth of insights and the scope of the findings. Finally, most studies did not conduct a socio-demographic analysis, thereby limiting an in-depth analysis of education and income disparities.

## 5. Conclusions

This scoping review highlights DMHIs for anxiety and depression in LATAM by emphasizing challenges and key success elements associated with implementation. In order to effectively implement DMHIs in LATAM, digital health policies should invest in digital proficiency training for healthcare workers, promoting participatory design to align DMHIs with workflows and cultural contexts, improving internet infrastructure, expanding digital access in underserved areas, and ensuring data privacy through regulations. Future research should explore strategies to boost clinician involvement, assess the sustainability and scalability of DMHIs across diverse contexts, and investigate prevention-focused MH interventions. Additionally, economic analyses comparing DMHIs’ cost-effectiveness with traditional methods would inform resource allocation in policy and practice. Additionally, collaboration across countries in LATAM to share best practices and policies will help to address MH disparities. The results of this scoping review serve as a call to action to advance LATAM-led research and policy involvement with DMHIs to improve not only the quality of these interventions but also their communities’ MH.

## Figures and Tables

**Table 1:** Search terms used in PubMed, APA PsycNet, Scielo, and LILACS.

Category	Search Terms Combined with AND
DHIs	“digital technology” OR “telemedicine” OR “digital interventions” OR “telehealth” OR “digital health” OR “internet-based” OR “e-mental health”
MH	“mental health” OR “Depression” OR “Anxiety”
Region	“Colombia” OR “Mexico” OR “Brazil” OR “Argentina” OR “Peru” OR “South America” OR “Central America” OR “Latin America”

**Table 2:** WHO DHI classification.

Author(s), Country, Year	Level 1	Level 2	Level 3
De la Cruz-Torralva, Escobar-Agreda, López, Amaro, Reategui-Rivera & Rojas-Mezarina, Peru, 2024 [20]	Interventions for persons	Targeted communication to persons; telemedicine	Consultations between remote person and healthcare provider; transmit targeted health information to person(s) based on health status or demographics
Zapata-Ospina et al., Colombia, 2024 [21]	Interventions for persons	Telemedicine, data management	Consultations between remote persons and healthcare providers
Araya et al., Peru and Brazil, 2021 [22]	Interventions for persons	Targeted communication to persons	Transmit targeted health information to person(s) based on health status or demographics
Lopes, da Rocha, Svacina, Meyer, Šipka & Berger, Brazil, 2023 [23]	Interventions for persons	Personal health tracking	Self-monitoring of health or diagnostic data by the individual
Dominguez-Rodriguez et al., Mexico, 2024 [24]	Interventions for persons	Personal health tracking	Self-monitoring of health or diagnostic data by the individual
Moretti et al., Brazil, 2024 [25]	Interventions for persons	Targeted communication to persons	Transmit untargeted health information to an undefined population
Martínez et al., Chile and Colombia, 2021 [26]	Interventions for persons	Targeted communication to persons	Simulated human-like conversations with individual(s); transmit targeted health information to person(s) based on health status or demographics; peer group for individuals; list health facilities and related information
Diez-Canseco, Peru, 2018 [27]	Interventions for healthcare providers	Healthcare provider decision support	Transmit targeted alerts and reminders to person(s)
Benjet et al., Colombia and Mexico, 2023 [28]	Interventions for persons	Personal health tracking	Self-monitoring of health or diagnostic data by the individual
Santa-Cruz et al., Peru, 2023 [29]	Interventions for persons	Targeted communication to persons	Simulated human-like conversations with individual(s); identify persons in need of services; manage referrals between points of service within the health sector
Daley, Hungerbuehler, Cavanagh, Claro, Swinton & Kapps, Brazil, 2020 [30]	Interventions for persons	On-demand communication with persons	Simulated human-like conversations with individual(s)
Martínez, Rojas, Martínez, Zitko, Irarrázaval, Luttges & Araya, Chile, 2018 [31]	Interventions for healthcare providers	Data management; healthcare provider decision support	Longitudinal tracking of a person’s health status and services; managing person-centered structured clinical records; data storage and aggregation
Alva-Arroyo, Ancaya-Martínez, & Floréz-Ibarra, 2021, Peru [32]	Interventions for persons	Telemedicine	Consultations between remote persons and healthcare providers
Pérez et al., Colombia, 2020 [33]	Interventions for persons	Telemedicine	Consultations between remote persons and healthcare providers
Gómez-Restrepo, Cepeda, Torrey, Castro, S., Uribe-Restrepo, Suárez-Obando & Marsch, Colombia, 2021 [34]	Interventions for healthcare providers	Healthcare provider decision support	Screen persons by risk or other health status

## Data Availability

No new data were created or analyzed in this study.

## Abbreviations

The following abbreviations are used in this manuscript:

CDISAH	Classification of Digital Interventions, Services, and Applications in Health
DHI	Digital Health Intervention
DMHI	Digital Mental Health Intervention
LATAM	Latin America
MH	Mental Health
mhGAP	Mental Health Gap Action Programme
RAPS	Rede de Atenção Psicossocial
WHO	World Health Organization

## Appendix A

**Table A1:** Coding used per database and per language.

English	Spanish	Portuguese
((“digital technology”[Title/Abstract] OR “telemedicine”[Title/Abstract] OR “digital interventions”[Title/Abstract] OR “telemedicine”[MeSH Terms] OR “digital health”[Title/Abstract] OR “internet-based”[Title/Abstract]) AND (((depression[Title/Abstract]) OR (anxiety[Title/Abstract])) OR (“mental health”[Title/Abstract]))) AND ((((((((colombia[MeSH Terms]) OR (mexico[MeSH Terms])) OR (argentina[MeSH Terms])) OR (brazil[MeSH Terms])) OR (Peru[Title/Abstract])) OR (central america[MeSH Terms])) OR (south america[Title/Abstract])) OR (latin america[MeSH Terms]))	((“Tecnología”[Title/Abstract] OR “telemedicina”[Title/Abstract] OR “digital”[Title/Abstract] OR “telemedicina”[MeSH Terms] OR “digital”[Title/Abstract] OR “internet”[Title/Abstract]) AND (((depresión[Title/Abstract]) OR (ansiedad[Title/Abstract])) OR (“salud mental”[Title/Abstract]))) AND ((((((((colombia[MeSH Terms]) OR (méxico[MeSH Terms])) OR (argentina[MeSH Terms])) OR (brasil[MeSH Terms])) OR (Perú[Title/Abstract])) OR (centroamérica[MeSH Terms])) OR (sudamérica[Title/Abstract])) OR (latinoamérica[MeSH Terms]))	((“tecnologia digital” OR “telemedicina” OR “intervenções digitais” OR “telessaúde” OR “saúde digital” OR “internet” OR “saúde mental digital”) AND (“saúde mental” OR “depressão” OR “ansiedade”)) AND (“Colômbia” OR “México” OR “Brasil” OR “Argentina” OR “Peru” OR “América do Sul” OR “América Central” OR “América Latina”)
Any Field: “digital technology” OR “telemedicine” OR “digital interventions” OR “telehealth” OR “digital health” OR “internet-based” OR “e-mental health” *AND* Any Field: “mental health” OR “Depression” OR “Anxiety” *AND* Any Field: “Colombia” OR “Mexico” OR “Brazil” OR “Argentina” OR “Peru” OR “South America” OR “Central America” OR “Latin America”	Any Field: “Colombia” OR “México” OR “Brasil” OR “Argentina” OR “Perú” OR “Sudamérica” OR “Centroamérica” OR “América Latina” OR “Latinoamérica” *AND* Any Field: “salud mental” OR “depresión” OR “ansiedad” *AND* Any Field: “tecnología digital” OR “intervenciones digitales” OR “telemedicina” OR “salud digital” OR “internet” OR “salud mental digital”	Any Field: “tecnologia digital” OR “telemedicina” OR “intervenções digitais” OR “telessaúde” OR “saúde digital” OR “internet” OR “saúde mental digital” *AND* Any Field: “saúde mental” OR “depressão” OR “ansiedade” *AND* Any Field: (“Colômbia” OR “México” OR “Brasil” OR “Argentina” OR “Peru” OR “América do Sul” OR “América Central” OR “América Latina”
(digital technology OR telemedicine OR digital interventions OR telehealth OR digital health OR internet-based OR e-mental health) AND (mental health OR depression OR anxiety) AND (Colombia OR Mexico OR Brazil OR Argentina OR Peru OR South America OR Central America)	(“Colombia” OR “México” OR “Brasil” OR “Argentina” OR “Perú” OR “Latinoamérica” OR “sudamérica” OR “centroamérica”) AND (“salud mental” OR “depresión” OR “ansiedad”) AND (“tecnología digital” OR “telemedicina” OR “salud digital” OR “internet”)-COVID-19-cyberbullying site:scielo.org)	(“tecnologia digital” OR “telemedicina” OR “intervenções digitais” OR “telessaúde” OR “saúde digital” OR “internet” OR “saúde mental digital”) AND (“saúde mental” OR “depressão” OR “ansiedade”) AND (“Colômbia” OR “México” OR “Brasil” OR “Argentina” OR “Peru” OR “América do Sul” OR “América Central” OR “América Latina”)
(“digital technology” OR “telemedicine” OR “digital interventions” OR “telehealth” OR “digital health” OR “internet-based” OR “e-mental health”) AND (“mental health” OR “Depression” OR “Anxiety”) AND (“Colombia” OR “Mexico” OR “Brazil” OR “Argentina” OR “Peru” OR “South America” OR “Central America” OR “Latin America”)	(“Colombia” OR “México” OR “Brasil” OR “Argentina” OR “Perú” OR “Sudamérica” OR “Centroamérica” OR “América Latina” OR “Latinoamérica” AND “salud mental” OR “depresión” OR “ansiedad” AND “tecnología digital” OR “intervenciones digitales” OR “telemedicina” OR “salud digital” OR “internet” OR “salud mental digital”)	(“tecnologia digital” OR “telemedicina” OR “intervenções digitais” OR “telessaúde” OR “saúde digital” OR “internet” OR “saúde mental digital”) AND (“saúde mental” OR “depressão” OR “ansiedade”) AND (“Colômbia” OR “México” OR “Brasil” OR “Argentina” OR “Peru” OR “América do Sul” OR “América Central” OR “América Latina”)

**Table A2:** Description of DMHIs in Latin America: barriers, facilitators, outcomes, and findings.

Author(s), Country, Year	Name of DMHI	MH Disorders	Target Population	Facilitators	Barriers	Study Design	Study Objective	Main Findings
1. De la Cruz-Torralva, Escobar-Agreda, López,. Amaro, Reategui-Rivera & Rojas-Mezarina, Peru, 2024 [20]	Mental Health Accompaniment Program (MHAP)	Depression, anxiety, substance abuse	Recently graduated physicians from the Universidad Nacional Mayor de San Marcos (*n* = 75)	Equal or better results than traditional methods High or increased patient satisfaction	No time-availability Lack of clinician involvement/management or organization resistance	Mixed methods study with follow-up	“This study aimed to evaluate the characteristics and the responses and perceptions of recently graduated physicians who work in rural areas of Peru as part of the Servicio Rural Urbano Marginal en Salud (Rural-Urban Marginal Health Service [SERUMS], in Spanish) toward a telehealth intervention to provide remote orientation and accompaniment in mental health”.	“The MHAP included 4 services: evaluation and screening, self-help, brief intervention, and suicide prevention”. “Implementation of the MHAP is effective in identifying and managing mental health conditions in newly graduated physicians working in rural areas of Peru. Although there is no evidence of digital mental health interventions for these professionals, our findings are consistent with other studies highlighting the usefulness of digital tools in identifying and treating mental health problems”.
2. Zapata-Ospina et al., Colombia, 2024 [21]	LivingLab Telehealth	Depression, anxiety, psychosis	Adults receiving telepsychology (*n* = 371) or telepsychiatry (*n* = 362)	DI is easier to access	Cost-effectiveness/insurance coverage More personalization/cultural appropriateness needed	Descriptive study with follow-up 8–15 days after initial care	“Describe the development and operation of the program and evaluate the degree of satisfaction of the patients”.	“Results of the triage are produced automatically by the medical records programme, which shows the patient’s risk and priority and thus allows interventions to be planned”. “Most of these patients (84.6%) considered the care they received on admission from the TPAH (pre-hospital care team member) to be useful or very useful, and considered that the quality of the telepsychology service was adequate”. “Of this sample, 199 (85.0%) reported that it was the first time they had ever received virtual care, and 69 (29.5%) had difficulty accessing mental health services in person, mainly due to financial problems, distant place of residence and transportation difficulties”.
3. Araya et al., Brazil and Peru, 2021 [22]	Smartphone app	Depression	Patients with clinically significant depressive symptoms with comorbid hypertension and/or Diabetes (*n* = 880 Brazil) (*n* = 432 Peru)	DI is easier to access Equal or better results than traditional methods	More training needed Too complicated/not understand technology Not enough literature/cannot be generalized	Two randomized control trials with 6-month follow-up	“To investigate the effectiveness of a digital intervention in reducing depressive symptoms among people with diabetes and/or hypertension”.	“In 2 RCTs of patients with hypertension or diabetes and depressive symptoms in Brazil and Peru, a digital intervention delivered over a 6-week period significantly improved depressive symptoms at 3 months when compared with enhanced usual care. However, the magnitude of the effect was small in the trial from Brazil and the effects were not sustained at 6 months”.
4. Lopes, da Rocha, Svacina, Meyer, Šipka & Berger, Brazil, 2023 [23]	Deprexis	Depression	General population with a high rate of depressive symptoms (*n* = 312)	Equal or better results than traditional methods DI is easier to access	High dropout rate	Randomized control trial	“Replication in Brazil previously reported effects of Deprexis on depressive symptom reduction; whether Deprexis is effective in reducing depressive symptoms and general psychological state in Brazilian users with moderate and severe depression in comparison with a control group that does not receive access to Deprexis”.	“Participants reported medium to high levels of satisfaction with Deprexis. These results replicate previous findings by showing that Deprexis can facilitate symptomatic improvement over 3 months. It also extends previous research by demonstrating that this intervention is effective in a Brazilian sample, which differs culturally and linguistically from the previously studied populations in Germany, Switzerland, and the United States”.
5. Dominguez- Rodriguezet al., Mexico, 2024 [24]	Mixed media (YouTube and PDFs) and IT-Lab	Depression, anxiety	General adult population (*n* = 36)	DI is easier to access Equal or better results than traditional methods DH is less costly	High dropout rate Too complicated/not understand technology Ensuring data storage safety	Randomized control trial, with follow-up after 3 and 6 months	“Aims to assess the efficacy of 2 modalities of a self-guided intervention (with and without chat support) in reducing various symptoms”	“For the SGWI group, symptom levels decreased compared to pre- and posttest. But for the SGWI+C group, the reduction in symptoms remained statistically relevant with minor to medium-sized effects for depression, widespread fear, and anxiety”. “Enhancing the utility of web-based interventions for reducing clinical symptoms by incorporating a support chat to boost treatment adherence seemed to improve the perception of the intervention’s usefulness”.
6. Moretti et al., Brazil, 2024 [25]	Online Self-Knowledge Journey	Depression	Adults in technical college (*n* = 9) Individuals in a public university (*n* = 18) Non-profit institute employees (*n* = 30) Platform used by adults (*n* = 2145) Private college students (*n* = 50)	High or increased patient satisfaction Increased training and support Personalized results	No time-availability Ensuring data storage safety High dropout rate	Mixed methods design	“To evaluate the platform’s user engagement and satisfaction in an understudied context”.	“Observed substantial dropout rates across all samples during the usage of the journey, as evidenced by the low completion rates”. “The overall satisfaction in samples 1–3 was high”. “The predominant reason cited for non-completion of the platform’s journey was a lack of time”.
7. Martínez et al., Chile and Colombia, 2021 [26]	Cuida tu Animo	Depression	Adolescents from two schools in each country (*n* = 199)	Increased training and support High or increased patient satisfaction, Equal or better results than traditional methods	High dropout rate More personalization/cultural appropriateness needed More training needed	Pilot mixed-methods study	“Evaluate the feasibility and acceptability and estimate the effects of an internet-based program for the prevention and early intervention of adolescent depression in high school students implemented in Santiago (Chile) and Medellín (Colombia)”.	“The results showed a change in depressive and anxious symptoms in Chilean adolescents, but no significant symptom changes in Colombian adolescents”. “Regarding the feasibility and acceptability, the program was used and accepted by the adolescents. Although the levels of satisfaction were moderate/high (62.1–82.5%), utilization rates were low overall”.
8. Diez-Canseco et al., Peru, 2018 [27]	mHealth	Depression, anxiety, psychosis, substance abuse, stress	Adult patients attending public primary health centers (*n* = 127) Primary health care providers (PHCPs) (*n* = 22)	Increased training and support	No time-availability Lack of clinician involvement/management or organization resistance More personalization/cultural appropriateness needed	Multi-phase design with follow-up	“To promote early detection, referral, and access to mental health care for patients with common mental disorders attending primary health care services”.	“With more than 750 screenings completed in real-world circumstances over a 9-week period, it shows the feasibility to integrate the mental health screening into primary care services as a routine procedure”. “Out of 10 patients, 7 actively sought specialized care, and 5 obtained a consultation with a mental health specialist, thus showing promising results for this type of intervention”.
9. Benjet et al., Colombia and Mexico, 2023 [28]	Silvercloud	Depression, anxiety	University students (*n* = 1319)	Reducing shame/stigma DH is less costly DI is easier to access High or increased patient satisfaction	Not useful for complex and severe cases Not enough literature/cannot be generalized	Randomized control trial with reassessment after 3 months	“Compare transdiagnostic self-guided and guided internet-delivered cognitive behavioral therapy (i-CBT) with treatment as usual (TAU) for clinically significant anxiety and depression among undergraduates in Colombia and Mexico”.	“Intent-to-treat analysis found significantly higher adjusted remission rates (ARD) among participants randomized to guided i-CBT than either self-guided i-CBT or TAU, but no significant difference between self-guided i-CBT and TAU”
10. Santa-Cruz et al., Peru, 2023 [29]	ChatBot-Juntos and remote Psychological First Aid (PFA)	Depression, anxiety, psychosis, substance abuse, stress, other	Underserved general population (*n* = 2027)	DI is easier to access DH is less costly Reducing shame/stigma	Lack of internet access More training needed	Retrospective cohort design with 3-month follow-up	“Describe the development, implementation, and participant outcomes of this innovative system of virtual mental health screening and support in the population of Lima affected during the COVID-19 pandemic”.	“Almost 80% of participants screened positive for psychological distress. 63% of people with psychological distress received PFA, and 32.1% of those were also referred for mental health care” “For those who were re-evaluated, significant improvements in SRQ score at 3 months post-intervention were seen in those participating in PFA”
11. Daley, Hungerbuehler, Cavanagh, Claro, Swinton & Kapps, Brazil, 2020 [30]	ChatBot-Vitalk	Depression, anxiety	General adult population (*n* = 3629)	DH is less costly DI is easier to access Time availability increased Reducing shame/stigma	High dropout rate	Primary evaluation of real-world data over a one-month period	“DHI: The goal of the conversations is to help the user reflect on experiences and learn techniques which can help them manage stress, mood and anxiety. A mood tracking tool helps to aid reflection”. “Study: To assess the engagement and effectiveness of Vitalk in reducing mental health symptoms (stress, anxiety, depression) over a one-month period”.	“Significant reduction in anxiety, depression, and stress scores with large effect sizes—Higher engagement correlated with better outcomes and also found to predict lower anxiety and depressive symptoms at follow up”. “Adult females under 24 years of age (52% 18–24 years, 76% female)”.
12. Martínez, Rojas, Martínez, Zitko, Irarrázaval, Luttges & Araya, Chile, 2018 [31]	Remote Collaborative Depression Care (RCDC) Program	Depression	Adolescents aged 13–19 (*n* = 143)	Time availability increased High or increased patient satisfaction Increased training and support	Lack of internet access More training needed Lack of clinician involvement/management or organization resistance Not useful for complex and severe cases	Two-group, cluster randomized clinical trial with a 12-week follow-up	“Tested the feasibility, acceptability, and effectiveness of a remote collaborative care program delivered by primary and specialized mental health teams to enhance the management of adolescent MDD in the Araucanía Region, an underserved area of Chile”.	“The RCDC intervention significantly surpassed enhanced usual care in terms of user satisfaction with psychological treatment. Phone monitoring was perceived as useful and provided them with innovative tools for the comprehensive management of adolescent depression”. “RCDC (at follow-up) for the treatment of adolescent depression did appear to have equivalent effectiveness compared with EUC, achieving comparable levels of depressive symptoms”
13. Alva-Arroyo, Ancaya-Martínez, & Floréz-Ibarra, 2021, Peru [32]	Internet HHV (Hospital Hermilio Valdizán), telehealth, and digital platforms	Depression, anxiety, psychosis, substance abuse, stress, other	Patients at Hermilio Valdizán Hospital (*n* = 4411)	Increased training and support	Ensuring data storage safety Lack of clinician involvement/management or culture organization resistance More training needed Not enough literature/cannot be generalized	Observation study	“The objective of this article is to present the telehealth experiences in a hospital specialized in mental health in Lima, Peru during the COVID-19 pandemic”.	“Concluded that the implementation of telehealth for the care of the users of the Hermilio Valdizán Hospital contributes to mental health care and reduces the gaps in access to specialized care in psychiatry due to the consequences of COVID-19”.
14. Pérez et al., Colombia, 2020 [33]	Telemedicine	Depression, anxiety, other	Patients enrolled for at least a year in a telemedicine program (*n* = 111)	Increased training and support	Lack of clinician involvement/management or culture organization resistance, More training needed	Descriptive study	“To describe the experience of physicians and patients in the Telepsychiatry programme at the University of Antioquia’s Faculty of Medicine in the first 12 months after its implementation in eight towns across Antioquia”.	“Most patients and health personnel are satisfied with telemedicine attention and found it of comparable quality to presencial modalities. However, they expressed concern on data confidentiality and low capacity of response in case of emergencies. Patients reported high satisfaction overall”.
15. Gómez-Restrepo, Cepeda, Torrey, Castro, Uribe-Restrepo, Suárez-Obando & Marsch, Colombia, 2021 [34]	Tablet-based screening system and Labbr (mobile app)	Depression, anxiety, psychosis, substance abuse	Patients attending primary care centers in urban and rural areas (*n* = 16,188)	Increased training and support	No time-availability Lack of clinician involvement/management or culture organization resistance	Modified stepped-wedge design with follow-up	“To improve the detection, diagnosis, and treatment of depression and unhealthy alcohol use through a technology-assisted model integrated into primary care”.	“Conducted a total of 22,354 screenings among 16,188 patients with a majority being female”. “10.1% prevalence of GP-confirmed depression and 1.3% of risky alcohol use; improved diagnosis rates due to integrated screening but faced challenges with time constraints”.
